# Exploring the Psychiatric Manifestations of Primary Sjögren's Syndrome: A Narrative Review

**DOI:** 10.1155/2024/5520927

**Published:** 2024-05-14

**Authors:** Mona Salehi, Azadeh Zamiri, Jeffrey Kim, Chenique Texeira, Ketki Shah, Sasidhar Gunturu

**Affiliations:** ^1^Department of Psychiatry, Bronx Care Health System, New York, NY, USA; ^2^Department of Psychiatry, Johns Hopkins University School of Medicine, Baltimore, MD, USA; ^3^Department of Psychiatry, University of Minnesota School of Medicine, Minneapolis, MN, USA; ^4^Department of Psychiatry, Icahn School of Medicine at Mount Sinai, New York, NY, USA

## Abstract

**Background:**

Primary Sjögren's syndrome (pSS) is recognized for its autoimmune origin. Its hallmark symptoms, dry eyes and mouth, result from glandular inflammation. Prior literature indicates that pSS not only affects the peripheral system but also involves the central nervous system (CNS), giving rise to various neuropsychiatric symptoms. However, there is limited published research on the psychiatric comorbidities in individuals with pSS.

**Methods:**

A comprehensive search was conducted on PubMed and Google Scholar for this narrative review. The search spanned from inception until August 2023. Its aim was to locate studies focusing on the psychiatric manifestations of pSS and the potential underlying mechanisms.

**Results:**

The most commonly reported psychiatric complications among these individuals are depression and cognitive dysfunction. Other psychiatric manifestations that have been reported in pSS individuals include anxiety, sleep disorders, psychosis, catatonia, bipolar disorder, and obsessive-compulsive disorder.

**Conclusion:**

In conclusion, patients with pSS often display multiple psychiatric symptoms. These symptoms can significantly impair functioning and reduce quality of life. Hence, prompt diagnosis and management are crucial.

## 1. Introduction

Sjögren's syndrome (SS) is an autoimmune disorder characterized by the infiltration of mononuclear cells and subsequent damage to the salivary and lacrimal glands. This syndrome can be either secondary Sjögren's syndrome, occurring with another autoimmune disease, or primary Sjögren's syndrome (pSS) when it manifests independently [1]. The prevalence of pSS varies across different populations and regions, but it is estimated to affect approximately 0.1% to 3% of the general population [2, 3]. The female-to-male ratio is around 9 : 1 [4]. Emerging evidence suggests that pSS can also affect the nervous system, leading to various psychiatric manifestations [5]. However, there is limited published research on the epidemiological aspects of these comorbidities among individuals with pSS. The prevalence of psychiatric manifestations in pSS varies widely across prior studies due to discrepancies in their methods [6–9]. It appears that psychiatric disorders are the primary manifestations of the disease. They do not appear to result from complications of the disease, its treatment, or a concurrent non-pSS-related event [10]. These conditions include various psychiatric disorders [11]. The comorbid psychiatric disorders include cognitive dysfunction [12], depression [13], bipolar mood disorder [14], psychosis [15, 16], catatonia [15, 17], and obsessive-compulsive disorder [11, 18]. Cognitive dysfunction, often called “brain fog,” is the most common psychiatric manifestation reported by pSS patients. This can affect attention, memory, and information processing speed [19]. Brain fog is usually followed by depression, which significantly impact patients' quality of life [6].

The precise underlying mechanism contributing to psychiatric involvement in pSS remains unknown. However, several potential mechanisms have been suggested. Antineuronal antibodies, such as those targeting the NR2 subunit of the NMDA receptor (anti-NR2 antibodies), antiribosomal P proteins found on neuronal surfaces (anti-P antibodies), and anti-aquaporin-4 (AQP4) antibodies, have been identified as potentially affecting the brain [12, 20, 21]. These antibodies are thought to contribute to the development of these conditions [20–23]. Other possible mechanisms are inflammation-related disturbances in central nervous system (CNS) [24], neurotransmitter dysregulation [25, 26], and ischemic damage caused by CNS vasculitis [11, 27].

Diagnosing these manifestations is challenging due to the lack of specific biomarkers and overlapping symptoms with other rheumatic and psychiatric disorders. Collaboration among rheumatologists, neurologists, and psychiatrists is crucial for accurate diagnosis and appropriate management [28]. Treatment strategies are multidimensional with the goal to alleviate symptoms, improve quality of life, and manage the underlying autoimmune process. Symptomatic treatment options include cognitive rehabilitation, antidepressant and anxiolytic medications, and pain management. Immunomodulatory therapies, such as corticosteroids, disease-modifying antirheumatic drugs, and biologic agents, should also be considered for patients [29, 30].

These manifestations significantly impact patients' quality of life [6]. They can also be the primary presenting symptoms of the disease in 50% to 80% of patients [31]. This can further complicate the diagnosis of pSS. Therefore, it is crucial to begin screening for these complications at disease onset and treat them adequately with the most effective options available.

In this paper, our aim is to review existing literature to enhance understanding of psychiatric aspects in pSS. We aim to shed light on the underlying mechanisms, clinical manifestations, diagnostic challenges, and treatment approaches.

## 2. Materials and Methods

We conducted a thorough search of PubMed and Google Scholar for this narrative review from the beginning until August 2023. Our aim was to find studies addressing psychiatric symptoms in pSS and their potential underlying mechanisms, without limiting based on publication dates. Only English language articles were considered during the review process. The search terms used included “Sjögren's syndrome,” “Sjögren,” “psychiatric manifestations,” “Psychiatric conditions,” “bipolar”, “obsessive-compulsive,” “obsession,” “anxiety disorder,” “depressive disorder,” “sleep disorder,” “catatonia,” “psychosis,” and “cognitive impairment.” Upon identification of each pertinent article, the reference list was explored to find additional relevant sources. Gray literature was not included in the search process.

## 3. Results

We found 1500 records through a database search, along with screening titles and abstracts. Upon reading the full texts, we excluded 1395 papers. In total, 105 full-text articles were included in this study.

The most common reported psychiatric complications among these individuals were depression and cognitive dysfunction. Other psychiatric manifestations that have been reported in pSS individuals include anxiety, sleep disorders, psychosis, catatonia, bipolar disorder, and obsessive-compulsive disorder (Box 1).

We extensively examined each of these psychiatric manifestations and their potential underlying pathophysiology, as well as their diagnosis and treatment approaches.

### 3.1. Sleep Disorders

Sleep disturbances are increasingly found to be an essential clinical presentation of rheumatologic disorders [32]. Pronounced sleep issues were found in pSS, with one study revealing that 75% of pSS patients reported moderate to severe sleep disruptions [33].

A recent meta-analysis underscored heightened sleep disruptions in pSS patients compared with controls. These disruptions encompass drowsiness, self-reported sleep problems, and frequent nocturnal awakenings [34]. Managing these disturbances is crucial, as they can significantly impact the quality of life in pSS patients [6, 35]. Accurate diagnosis and proper treatment of these sleep problems could also relieve associated symptoms like pain, mood swings, and fatigue, which leads to improved physical and cognitive performance and overall well-being [34]. Using a standardized sleep questionnaire, Gudbjörnsson et al. found a significantly greater sleep deficit (defined as the difference between required and actual sleep time) in pSS patients compared to healthy controls and rheumatoid arthritis (RA) patients [36]. This study revealed significant difficulties in sleep initiation and maintenance in pSS patients.

The sleep disturbances in pSS are related to several factors. These factors may overlap with symptoms of fibromyalgia [33]. They also relate to mood disorders [37], muscle and joint pain, and sleep interruptions due to hydration needs from sicca symptoms [38]. Restless leg syndrome and coexisting obstructive sleep apnea (OSA) are also more prevalent in pSS patients and cause the sleep problems [39]. A recent case-control study in Italy evaluated the sleep quality in 29 female patients with pSS using Pittsburgh Sleep Quality Index (PSQI) [35]. They found reduced sleep quality in 83% of pSS patients, which was correlated with mood disturbances, as well as low levels of physical and mental health in these patients [35].

Effective management of sleep disruption needs a comprehensive approach. Treating the underlying autoimmune inflammation is necessary, often achieved through immunosuppressive medications [40]. Additionally, it is crucial to address dryness-related symptoms, which are a hallmark of pSS. Moisturizing therapy for the eyes and mouth can alleviate discomfort, thereby promoting more restful sleep [38]. Moreover, cognitive behavioral therapy for insomnia (CBT-I) has shown promise in improving sleep quality by modifying maladaptive sleep behaviors and thought patterns [41].

### 3.2. Psychosis and Catatonia

While peripheral nervous system (PNS) disease is a well-established complication of pSS, this disorder's CNS complications have received relatively little attention until recently [42]. Although CNS involvement may be infrequent, it remains a significant complication. CNS involvement can manifest with focal symptoms like motor, sensory, or speech disturbances. It can also present with nonfocal neurological symptoms such as cognitive dysfunction and various psychiatric disorders [7]. Spezialetti et al. evaluated a group of 77 patients with both secondary SS and pSS who also had CNS involvement and reported psychiatric and cognitive dysfunction in over 80% of these patients, with psychosis occurring in 8% [12]. While comorbid depression is commonly reported in patients with pSS, comorbid psychosis is a relatively rare condition [12, 16].

Catatonia manifests as a range of physical and behavioral symptoms that arise from various psychiatric, neurological, or medical sources. This condition, which affects motor functioning, behavior, and emotional responses, is associated with numerous underlying factors [43]. A recent meta-analysis revealed a mean prevalence of catatonia to be 9.0%. The highest prevalence was observed in the presence of medical or neurological illness without comorbid psychiatric conditions (20.6%), while the lowest prevalence was found in mixed psychiatric samples (5.7%) [44]. The prevalence of catatonia in rheumatologic patients has not been extensively studied and documented to date. However, there are a few case studies highlighting its manifestation in rheumatologic disorders. One of the most prevalent occurrences is in anti N-methyl-D-aspartate receptor (NMDAR) encephalitis, with an estimated prevalence of 64% [45]. A significant number of conditions that make individuals prone to catatonia demonstrate connections with neuroinflammation. Neuroinflammation, characterized by microglial activation and the release of proinflammatory cytokines, disrupts neurotransmitter balance and synaptic functioning. This disruption contributes to the motor abnormalities and cognitive deficits observed in catatonia [46]. Immune system dysregulation, including autoimmunity and elevated levels of inflammatory markers, has been noted in individuals with catatonic features. This further underscores the link between the immune response and neuropsychiatric manifestations [45].

Psychosis is a mental health condition characterized by a disconnection from reality, with symptoms including hallucinations, delusions, disorganized thinking, and impaired insight. It can manifest as a primary feature of psychotic disorders like schizophrenia or as a symptom of other mental health conditions, neurological and medical disorders, or substance abuse [47]. A recent meta-analysis found the median lifetime prevalence of psychosis to be 7.49 per 1000 [48]. The majority of epidemiological studies have established a broad association between autoimmunity and psychotic disorders [49, 50]. In a Danish nationwide study, the risk of psychosis following an autoimmune disease diagnosis was found to increase by 45% [51]. Additionally, a recent meta-analysis by Cullen et al. [49] revealed that a diagnosis of a non-neurological autoimmune disease raised the risk of subsequent psychotic disorder diagnosis by 43%.

There are limited case studies on the presence of psychosis and catatonia in pSS [16, 23, 52–55]. Raps et al. presented a case of a 47-year-old man admitted due to psychosis, previously diagnosed with schizophrenia 18 years ago. During admission, pSS was clinically and historically diagnosed, and an unexplained temporal relationship between psychotic flare-ups and highly elevated erythrocyte sedimentation rates (ESR) was noted [53]. Two case studies reported female adolescents with new onset psychosis which did not typically respond to antipsychotic medications. Further investigations confirmed a diagnosis of pSS [16, 55]. Interestingly, rituximab infusion resulted in an improvement of psychiatric symptoms in these patients. This suggests that early consideration of rituximab as a treatment option for pSS-associated psychiatric disturbances may be warranted [16, 55]. Interestingly, rituximab infusion led to an improvement of psychiatric symptoms in these patients, indicating that early consideration of rituximab as a treatment option for pSS-associated psychiatric disturbances may be warranted [55]. Moreover, Moll et al. managed similar psychotic symptoms by prescribing oral prednisone. Remarkably, within a week, the patient had a considerable reduction in visual hallucinations and paranoid ideation, alongside an increased IQ [23].

Only two case studies reported catatonia and psychosis in pSS patients [16, 54]. Rosado et al. documented the case of a 21-year-old African female with psychosis. She initially received risperidone but later experienced a catatonic state characterized by negative mood, mutism, immobility, and refusal to eat or drink, along with fever. Further investigations revealed lymphadenopathy, bicytopenia, and elevated inflammatory markers. A salivary gland biopsy confirmed the diagnosis of pSS. The patient's psychiatric symptoms improved upon discontinuation of antipsychotic medications and the initiation of immunosuppressive therapy with hydroxychloroquine and prednisolone [54]. Similarly, Sivakumar et al. reported a 19-year-old female with acute onset psychosis and catatonia whose symptoms worsened with antipsychotic medications. She was later diagnosed with pSS, and her psychiatric symptoms gradually improved on immunomodulators [16]. Previous literature also reported instances where immunosuppressants such as azathioprine, methotrexate, and cyclosporine yielded positive outcomes in the treatment of pSS-related CNS involvements [56]. It is important to note that psychotropic medications should be administered cautiously in patients with autoimmune diseases. Some of these drugs, such as chlorpromazine, carbamazepine, and lithium carbonate, are known to potentially trigger autoimmune-like conditions [57].

As emphasized in these studies, new-onset psychosis can be the initial manifestation of pSS. Therefore, it is crucial to consider pSS in the differential diagnosis when assessing patients with these emergent symptoms, especially if they do not respond typically to psychotropic medications.

### 3.3. Obsessive-Compulsive Disorder

Obsessive-compulsive disorder (OCD) is a mental health condition characterized by intrusive, distressing thoughts (obsessions) and repetitive behaviors or mental acts performed to reduce the anxiety associated with these obsessions (compulsions). These behaviors can significantly impair a person's daily functioning and quality of life [58]. OCD can manifest as an initial symptom before the more commonly recognized symptoms of pSS appear [59, 60]. Wang et al. conducted a cohort study on a nationwide sample of patients with systemic autoimmune diseases including pSS. They found an increased risk of OCD in these populations with a hazard ratio of 2.38 in pSS patients [61]. Moreover, in a study of 103 pSS patients by Karaiskos et al. [62], 27.2% displayed traits of obsessiveness in their personalities. This heightened prevalence of OCD can significantly affect overall well-being and contribute to increased distress among these individuals [61].

Only a few studies have explored the connection between pSS and OCD. In one case report, a 17-year-old female with treatment-resistant OCD and depression was later diagnosed with pSS during her hospitalization. Her OCD symptoms completely disappeared after initiating immunotherapy with plasmapheresis and intravenous methylprednisolone [59]. Similarly, De Carvalho and Ribeiro described a case of a 40-year-old woman who initially presented with dry mouth and eyes alongside behavioral changes. She was diagnosed with OCD and treated with fluoxetine, risperidone, and later aripiprazole. However, her laboratory tests revealed positive autoimmune markers for pSS, and diagnostic tests confirmed it. She was started on hydroxychloroquine which completely controlled her OCD symptoms without psychiatric medications [63].

There are several possible mechanisms regarding the link between OCD and pSS. Some studies suggest that cytokines found in pSS may cross the blood-brain barrier, potentially causing OCD through various processes such as causing abnormalities in serotonergic signaling [64, 65]. Additionally, pSS could exacerbate OCD symptoms. This may occur in individuals with subclinical OCD vulnerability. It could happen directly by affecting the biological basis of OCD. Alternatively, it could occur indirectly by worsening vulnerabilities to depression or other anxiety disorders [66]. Another hypothesis suggests a shared genetic or epigenetic basis between pSS and OCD. This is similar to observed features in other autoimmune diseases associated with OCD, like rheumatic fever [67]. Future research should aim at documenting more cases of individuals with both pSS and OCD diagnoses. Additionally, it should investigate the role of immunosuppressive drugs in managing OCD symptoms.

### 3.4. Bipolar Disorder

Bipolar disorder (BD) is a complex mental health condition characterized by extreme mood swings. These mood swings can fluctuate between periods of elevated and irritable mood (mania or hypomania) and depressive episodes [68]. Previous studies have shown that systemic autoimmune diseases including pSS are associated with an increased risk of BD [14, 69]. However, research on the connection between BD and pSS remains limited.

Chebli et al. [70] presented a 54-year-old female who was diagnosed with pSS and was started on corticosteroids. Despite treatment, she still experienced mood disturbances. As a result, she was referred to a psychiatric ward. There, she received a diagnosis of bipolar disorder (BD) and histrionic personality disorder. Treatment with mood stabilizers and prednisone resulted in significant improvement within four weeks. This resolved her mood instability and agitation. Another study reported a 43-year-old male presented with neuropsychiatric complaints, such as elevated mood, increased energy, and headaches. Examination indicated xerophthalmia and xerostomia, leading to a diagnosis of pSS. MRI evaluation showed periventricular white matter lesions on T2-weighted images. Additionally, a psychiatric evaluation diagnosed the patient with BD, which was effectively treated with carbamazepine and azathioprine [71]. It is important to note that while pSS-associated psychiatric symptoms may respond well to immunosuppressive therapy, caution is warranted when considering corticosteroids. This is because corticosteroids have the potential to trigger psychiatric symptoms such as manic episodes and psychosis [72].

The relationship between bipolar disorder and immune diseases is bidirectional, with prior research indicating a significant rise in the occurrence of immune diseases among individuals with BD [73]. However, the underlying pathophysiological mechanisms remain unclear. Patients with both BD and autoimmune diseases tend to have a lower average life expectancy, a higher risk of self-injury, increased readmission rates, and face more challenging treatment and management. This includes a higher risk of in-hospital death [70, 74]. Therefore, timely diagnosis and management of BD in pSS patients are crucial.

### 3.5. Anxiety and Depression

Depression manifests as persistent sadness, lack of interest or fatigue, low energy, and guilt. It can negatively affect individuals' function and decrease their quality of life [75]. It has been shown that depression is common among patients with chronic diseases, including cancer, cardiovascular disease, diabetes, arthritis, and autoimmune diseases [76]. Previous studies showed that the prevalence of depression among pSS patients could be as high as 32% to 46% [77–79]. According to a systematic review and meta-analysis by Wan et al., the prevalence of depression and anxiety is approximately threefold higher in dry eye disorder (DED) patients compared to healthy controls. The difference was irrespective of the etiology of DED. However, data showed that patients with DED due to pSS suffer from more severe depression and anxiety [80].

**Figure 1:**
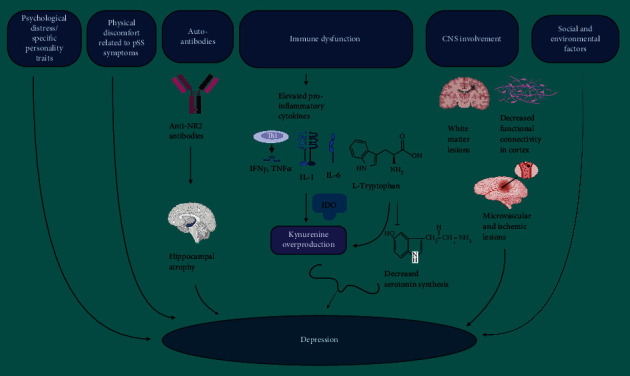
Possible mechanisms underlying depression in primary Sjögren's syndrome (pSS). (1) General psychological distress and specific personality traits like neuroticism, psychoticism, and obsessiveness might reduce stress tolerance and lead to depression. (2) Physical discomfort of pSS including fatigue, cognitive symptoms, sicca symptoms, and autonomic nervous system issues could contribute to depressive symptoms. (3) Elevated serum antibodies against N-methyl aspartate receptor (NMDAR) subtype NR2 found in pSS patients are associated with hippocampal atrophy, contributing to cognitive impairment and mood disorders in these patients. (4) Elevated levels of cytokines such as interferon-*γ* (IFN-*γ*), interleukin 1 (IL-1), interleukin 6 (IL-6), and tumor necrosis factor alpha (TNF-*α*) in pSS can trigger the overproduction of kynurenine and its metabolites from tryptophan through the indoleamine 2,3-dioxygenase (IDO) enzyme, diverting tryptophan away from serotonin production in the central nervous system (CNS). This imbalance in the hippocampus leads to depression, slower cognitive function, and other cognitive disorders. (5) Frequent central nervous system (CNS) white matter lesions (WML) were found in pSS, which can be linked to depression. Moreover, microstructural changes and decreased functional connectivity in the somatosensory cortex and corticospinal tract, as well as microvascular and ischemic lesions causing functional impairment in brain regions, have been observed in pSS. (6) Negative social and environmental factors, such as increased economic burden and lack of social support, may exacerbate depressive tendencies. Abbreviations: pSS: primary Sjögren's syndrome; TNF-*α*: tumor necrosis factor alpha; IL-6: interleukin 6; IL-1: interleukin 1; IFN- *γ*: interferon-*γ*; Anti-NR2: serum antibodies against N-methyl aspartate receptor (NMDAR) subtype NR2; IDO: indoleamine 2,3-dioxygenase enzyme; CNS: central nervous system. (This figure is created by Bioreneder.com).

While the precise mechanisms underlying depression and anxiety in pSS patients remain unclear, there are several possible mechanisms that can be considered (Figure 1).

*General Psychological Distress and Personality Traits*. Elevated levels of general psychological distress [81] and specific personality traits like neuroticism, psychoticism, and obsessiveness [82] might reduce stress tolerance.

*Physical Discomfort of pSS*. The physical discomfort associated with pSS, including fatigue, cognitive symptoms, sicca symptoms, and autonomic nervous system issues could contribute to depressive symptoms [83].

*Social and Environmental Factors*. Negative social and environmental factors, such as increased economic burden and lack of social support, may exacerbate depressive tendencies [76].

*Common Immune Dysfunction/Inflammation*. Shared immune dysfunction between pSS and depression has been observed. Inflammation has been identified as a mediating pathway for both the risk and neuroprogression of depression [84, 85]. Proinflammatory cytokines such as interleukin 1 (IL-1) and interleukin 6 (IL-6) have been implicated in both pSS and depression [40, 86, 87].

*Neurotransmitters Dysregulations*. Elevated levels of cytokines such as interferon-*γ* (IFN-*γ*), IL-1, and tumor necrosis factor alpha (TNF-*α*) in pSS can trigger the overproduction of kynurenine and its metabolites from tryptophan through indoleamine 2,3-dioxygenase (IDO) enzyme. This diverts tryptophan away from serotonin production in the CNS [25, 26]. This imbalance in the hippocampus leads to depression, slower cognitive function, and other cognitive disorders [88]. The affected cells, including microglia and astrocytes, experience reduced glutamate reuptake and increased glutamatergic signaling. This results in decreased serotonin production and can induce nociceptive and depressive behaviors. This cascade of effects can also perpetuate inflammation [26, 64, 89].

*CNS Involvement*. Frequent central nervous system white matter lesions (WML) were found in pSS [90] which can be linked to depression. Additionally, microstructural changes and decreased functional connectivity have been noted in the somatosensory cortex and corticospinal tract [91]. Furthermore, microvascular and ischemic lesions causing functional impairment in brain regions have been observed in pSS [27].

*Anti-NR2 Antibodies and Hippocampal Atrophy*. Elevated serum antibodies against N-methyl aspartate receptor (NMDAR) subtype NR2 were found in more pSS patients with depression than nondepressed individuals [20]. Positive anti-NR2 antibodies are also associated with hippocampal atrophy in pSS, contributing to cognitive impairment and mood symptoms [92].

In summary, the connection between pSS and depression involves various complex factors, including psychological, physiological, immunological, and neurostructural aspects.

Different studies have conflicting views on the impact of certain anti-inflammatory drugs, like IL-1 receptor antagonists and rituximab, on depressive disorder and mental fatigue in pSS patients [93, 94]. To address depression and mental fatigue, supplementing the medication regimen with antianxiety and antidepressant medications like paroxetine is recommended [76]. However, it is important to note that antidepressants and anxiolytics might induce dry eye disease due to their anticholinergic effects [95]. While both selective serotonin reuptake inhibitors (SSRIs) and serotonin and norepinephrine reuptake inhibitors (SNRIs) carry a risk of dry eye disease, SSRIs tend to result in lower Schirmer scores [96]. One nonmedication treatment modality in this group could be exercise therapy, such as a walking program. This approach can reduce muscle tension, depression, and fatigue by increasing endorphins and providing microelectrical stimulation in the nervous system [97]. Psychotherapy and behavioral interventions are also suggested to improve the patient's understanding of their disease and mitigate negative emotions [98]. Moreover, traditional Chinese medicine (TCM) has also shown promise in alleviating anxiety and depressive symptoms in pSS patients in recent studies [99, 100].

### 3.6. Cognitive Dysfunction

Cognitive dysfunction, often called “brain fog,” is one of the most common neuropsychiatric manifestations reported by pSS patients. It can affect attention, memory, and information processing speed [101]. The clinical presentations of cognitive dysfunction in pSS include difficulty concentrating, memory issues, slowed thinking or cognitive processing, mental fatigue, word-finding problems, and decreased multitasking abilities [101]. The prevalence of cognitive dysfunction in pSS patients varies between 44% and 100% [6, 13, 79, 102].

Previous studies have found that the majority of cognitive deficits in pSS may result from fronto-subcortical dysfunction. These deficits tend to remain relatively stable over time, with only rare instances of progression to dementia [103, 104]. Additionally, cognitive dysfunction has been associated with pain, depression, fatigue, and lower quality of life in pSS patients [78, 79].

The exact cause of cognitive deficits in pSS is not fully understood. However, a strong association has been found between cognitive deficits in pSS and the presence of anti-SSA (anti-Sjögren's syndrome-related antigen A) autoantibodies and magnetic resonance spectroscopy (MRS) alterations. This association persists regardless of an individual's age or gender. These studies found that despite the late onset of pSS, cognitive issues appear to be more common than age-related cognitive decline. This could potentially be due to subclinical inflammatory damage rather than structural microvascular damage, such as white matter lesions seen in cerebrovascular disease [79, 103]. Diagnostic and treatment challenges arise due to the varied neurological manifestations in pSS. However, previous studies emphasize on using MRI scans to identify underlying causes and assess the potential benefits of immunomodulatory treatments [79, 105].

## 4. Conclusions

In conclusion, pSS patients frequently exhibit various psychiatric manifestations including anxiety, sleep disorders, psychosis, catatonia, bipolar disorder, cognitive dysfunction, and obsessive-compulsive disorder. These psychiatric manifestations can be the primary presenting symptoms of pSS causing significant functional impairment and reduced quality of life. Therefore, timely diagnosis and management of these conditions are crucial.

## Figures and Tables

**Box 1:**
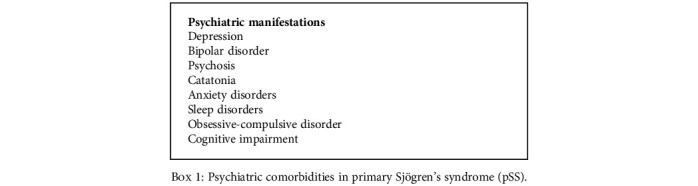
Psychiatric comorbidities in primary Sjögren's syndrome (pSS).
